# Amino-Terminal Processing of *Helicobacter pylori* Serine Protease HtrA: Role in Oligomerization and Activity Regulation

**DOI:** 10.3389/fmicb.2018.00642

**Published:** 2018-04-16

**Authors:** Nicole Albrecht, Nicole Tegtmeyer, Heinrich Sticht, Joanna Skórko-Glonek, Steffen Backert

**Affiliations:** ^1^Division of Microbiology, Department of Biology, Friedrich-Alexander-Universität Erlangen-Nürnberg, Erlangen, Germany; ^2^Division of Bioinformatics, Institute of Biochemistry, Friedrich-Alexander-Universität Erlangen-Nürnberg, Erlangen, Germany; ^3^Department of General and Medical Biochemistry, Faculty of Biology, University of Gdańsk, Gdańsk, Poland

**Keywords:** *C. jejuni*, H. pylori, HtrA, secretion, chaperone, E-cadherin, molecular pathogenesis, virulence

## Abstract

The HtrA family of serine proteases is found in most bacteria, and plays an essential role in the virulence of the gastric pathogen *Helicobacter pylori*. Secreted *H. pylori* HtrA (HtrA*^Hp^*) cleaves various junctional proteins such as E-cadherin disrupting the epithelial barrier, which is crucial for bacterial transmigration across the polarized epithelium. Recent studies indicated the presence of two characteristic HtrA*^Hp^* forms of 55 and 52 kDa (termed p55 and p52, respectively), in worldwide strains. In addition, p55 and p52 were produced by recombinant HtrA*^Hp^*, indicating auto-cleavage. However, the cleavage sites and their functional importance are yet unclear. Here, we determined the amino-terminal ends of p55 and p52 by Edman sequencing. Two proteolytic cleavage sites were identified (H46/D47 and K50/D51). Remarkably, the cleavage site sequences are conserved in HtrA*^Hp^* from worldwide isolates, but not in other Gram-negative pathogens, suggesting a highly specific assignment in *H. pylori*. We analyzed the role of the amino-terminal cleavage sites on activity, secretion and function of HtrA*^Hp^*. Three-dimensional modeling suggested a trimeric structure and a role of amino-terminal processing in oligomerization and regulation of proteolytic activity of HtrA*^Hp^*. Furthermore, point and deletion mutants of these processing sites were generated in the recently reported *Campylobacter jejuni* Δ*htrA*/*htrA^Hp^* genetic complementation system and the minimal sequence requirements for processing were determined. Polarized Caco-2 epithelial cells were infected with these strains and analyzed by immunofluorescence microscopy. The results indicated that HtrA*^Hp^* processing strongly affected the ability of the protease to disrupt the E-cadherin-based cell-to-cell junctions. Casein zymography confirmed that the amino-terminal region is required for maintaining the proteolytic activity of HtrA*^Hp^*. Furthermore, we demonstrated that this cleavage influences the secretion of HtrA*^Hp^* in the extracellular space as an important prerequisite for its virulence activity. Taken together, our data demonstrate that amino-terminal cleavage of HtrA*^Hp^* is conserved in this pathogen and affects oligomerization and thus, secretion and regulatory activities, suggesting an important role in the pathogenesis of *H. pylori*.

## Introduction

Infection by the Gram-negative pathogen *Helicobacter pylori* can cause chronic, mostly asymptomatic, gastritis, whereas more severe gastric diseases including adenocarcinoma, mucosa-associated lymphoid tissue (MALT) lymphoma and peptic ulceration occur less often (Polk and Peek, 2010). In fact, about half of the human world population carries these bacteria, and the majority of patients are colonized persistently by *H. pylori* (Pachathundikandi et al., 2016; Smolka and Schubert, 2017). The interplay between several bacterial, host and environmental factors is crucial for the virulence of the pathogen, largely influencing the clinical outcome of an infection (Servetas et al., 2016; Figueiredo et al., 2017; Gobert and Wilson, 2017; Shimizu et al., 2017). Besides the two well-known bacterial pathogenicity factors, the vacuolating toxin (VacA) associated with cellular vacuolation, apoptosis or immune cell inhibition, the cytotoxin-associated genes pathogenicity island (*cag*PAI), which encodes a type IV secretion system (T4SS) for the delivery of the CagA protein across the bacterial membrane into the host cell, additional *H. pylori* determinants are important for virulence (Backert and Clyne, 2011; Bridge and Merrell, 2013; Naumann et al., 2017). The FlaA protein, which is involved in flagella-mediated motility, the urease being essential for neutralizing the acidic pH in the human stomach and inflammasome activation, as well as various adhesins are important determinants for the virulence of *H. pylori* (Salama et al., 2013; Koch et al., 2015; Pachathundikandi et al., 2015; Javaheri et al., 2016; Bugaytsova et al., 2017). Furthermore, the serine protease high-temperature requirement A protein of *H. pylori* (HtrA*^Hp^*) was recently identified as a novel secreted virulence factor, which can target various host cell surface proteins (Hoy et al., 2010, 2012; Backert et al., 2016; Schmidt et al., 2016a,b; Tegtmeyer et al., 2016, 2017).

In many bacterial pathogens, HtrA proteases are widely conserved and play an important role in the virulence and survival of microbes under stress conditions (Hoy et al., 2013; Backert et al., 2017; Posselt et al., 2017; Skorko-Glonek et al., 2017). In addition to the protease function, HtrA acts as a chaperone being responsible for protein quality control and degradation of misfolded proteins in the periplasm (Kim and Kim, 2005; Hoy et al., 2013), suggesting that HtrA is highly active under harsh conditions (Hoy et al., 2013). In Gram-negative bacteria, HtrA proteases are actively transported in the periplasmic space and form proteolytic active oligomers (Singh et al., 2011; Skorko-Glonek et al., 2017). However, the mature form of HtrA in *E. coli* (HtrA*^Ec^*; 48 kDa) produces two 43 kDa polypeptides by partial auto-cleavage. This process seems to be positively stimulated under reducing conditions by substrates or peptides resulting from degraded HtrA products (Skórko-Glonek et al., 2003; Jomaa et al., 2009). Recent studies have shown that HtrA from the gastrointestinal pathogens *H. pylori* and *Campylobacter jejuni* can be actively secreted into the extracellular space (Löwer et al., 2008; Hoy et al., 2010, 2012; Boehm et al., 2012; Abfalter et al., 2016; Schmidt et al., 2016b; Wessler and Backert, 2018). For *H. pylori* and *C. jejuni* it was demonstrated that secreted and recombinant HtrA can cleave various host cell proteins such as the ectodomain of the cell-to-cell adhesion protein E-cadherin (Hoy et al., 2010, 2012; Boehm et al., 2012; Schmidt et al., 2016b). In addition, HtrA*^Hp^* cleaves fibronectin (Hoy et al., 2010) and the tight junction proteins occludin and claudin-8 (Tegtmeyer et al., 2017). In case of E-cadherin, cleaving-off the extracellular domain by HtrA can open the cell-to-cell junctions in the host cell monolayer and, thus, promote paracellular transmigration of both bacterial pathogens (Boehm et al., 2012; Hoy et al., 2012). By infecting polarized Caco-2 or MKN-28 cells, respectively, we were able to show that overexpression of HtrA*^Hp^* in *H. pylori* led to an increased damage of cell-to-cell junctions and significant decreased TER-values over time (Harrer et al., 2017). In addition, the transepithelial migration by *H. pylori* was elevated as a result of increased HtrA expression (Harrer et al., 2017). Moreover, a crucial role of HtrA in the immunopathology and induction of host cell apoptosis in the gut was demonstrated by infecting mice with *C. jejuni* (Heimesaat et al., 2014a,b). For *H. pylori* it was shown that an elevated expression of HtrA is associated with an increased proteolytic activity that led to a positive regulation of T4SS-dependent translocation and phosphorylation of CagA in epithelial cells (Harrer et al., 2017). Furthermore, the generation of *htrA* knockout mutants for about hundred worldwide *H. pylori* isolates failed so far, suggesting an outstanding importance of HtrA*^Hp^* for yet unknown cellular processes of *H. pylori* (Salama et al., 2004; Hoy et al., 2010; Tegtmeyer et al., 2016). In line with these observations, we have demonstrated that specific pharmacological inhibition of the HtrA protease activity killed *H. pylori* effectively, whereas the growth of other pathogens such as *Salmonella* or *Shigella* was not affected (Tegtmeyer et al., 2016).

Generally, HtrA proteins are composed of an amino-terminal signal peptide, which is followed by the trypsin-like protease domain and finally one or two carboxy-terminal PDZ domains (Gottesman et al., 1997; Frees et al., 2013). Three HtrA homologs, DegS, DegQ, and DegP, are expressed by *E. coli* representing the best-characterized HtrA model systems (Singh et al., 2011; Skorko-Glonek et al., 2013). While DegP works as an ATP-independent chaperone protease, DegS is characterized as a regulatory protease (Bass et al., 1996; Ruiz and Silhavy, 2005; Jiang et al., 2008). However, DegQ functions as a pH-related protein and chaperone, which is involved in protein quality control. Thus, this protease plays a very important role under acid stress conditions (Bai et al., 2011; Sawa et al., 2011; Malet et al., 2012). We have shown that the *htrA* gene is present in more than one thousand *H. pylori* strains from Europe, Asia, North and South America as well as Australia, and the HtrA*^Hp^* protein sequence is conserved within these isolates (Tegtmeyer et al., 2016). Analysis of bacterial lysates by Western blotting indicated that HtrA*^Hp^* is expressed as a double-band with molecular weights of 52 kDa (p52) and 55 kDa (p55), respectively. In addition, recombinant HtrA*^Hp^* showed this characteristic double-band, suggesting processing of the protein (Tegtmeyer et al., 2016). Here, we aimed to investigate the function of HtrA*^Hp^* processing and to identify the involved protease. Even though active secretion of HtrA*^Hp^* in the extracellular space might be conserved among various *H. pylori* isolates, the underlying mechanism is currently not understood (Tegtmeyer et al., 2016). Thus, we also investigated the role of HtrA*^Hp^* processing during protein secretion and proteolytic activity regulation.

## Materials and Methods

### *H. pylori, E. coli*, and *C. jejuni* Growth

*Helicobacter pylori* strains 35A, 26695, and P12 were used for Edman sequencing. Generally, *H. pylori* was grown at 37°C for 48–72 h on GC agar (Oxoid, Wesel/Germany) plates supplemented with 10% donor horse serum (Biochrom AG, Berlin/Germany), 1% vitamin mix, 10 μg/mL vancomycin, 1 μg/mL nystatin, and 5 μg/mL trimethoprim (Kumar Pachathundikandi et al., 2011; Wiedemann et al., 2012). The *C. jejuni* Δ*htrA*/*htrA^Hp^* complementation system was applied to mutagenize the amino-terminal cleavage sites of HtrA*^Hp^* (Boehm et al., 2017). The corresponding isogenic knockout mutant *C. jejuni* 81–176Δ*htrA* was characterized earlier (Brøndsted et al., 2005; Baek et al., 2011; Boehm et al., 2012). Commonly, the *C. jejuni* strains were grown at 37°C for 48–72 h on Campylobacter blood-free selective agar base containing selective supplement. If required for mutants, 20 μg/mL of chloramphenicol and/or 30 μg/mL kanamycin were added. All antibiotics were obtained from Sigma-Aldrich (St. Louis, MO/United States). Both *C. jejuni* and *H. pylori* were cultured under microaerobic conditions produced by CampyGen packs in 2.5 L anaerobic jars (Oxoid) (Patel et al., 2013; Tenguria et al., 2014). Chemically competent One Shot^TM^ TOP-10 *E. coli* (Invitrogen, Darmstadt/Germany) were grown in LB broth medium consisting of 10 g/L tryptone, 5 g/L yeast extract, and 10 g/L NaCl.

### Amino-Terminal Sequencing of *H. pylori* HtrA

Edman sequencing of proteins was performed on blotted proteins on a PVDF membrane using the ABI Procise 494 sequencer. HtrA fragments were eluted and amino-terminal sequencing done by Alphalyse A/S (Odense/Denmark).

### Mutagenesis of *H. pylori htrA* and Genetic Complementation in *C. jejuni*

For expression of the amino-terminal tagged GST-fusion protein, the pGEX-6P-1 plasmid encoding the HtrA*^Hp^* of strain 26695 (without signal peptide) was applied in this study (Löwer et al., 2008). This construct was a kind gift from Silja Wessler (University of Salzburg/Austria) and used as template for the generation of amino-terminal deleted variants of *H. pylori* 26695 HtrA. The mutagenesis PCR was performed using Platinum^TM^ *Taq* DNA Polymerase High Fidelity (High Fidelity buffer, Thermo Fisher Scientific, Darmstadt/Germany) with 400 ng template DNA following the manufacturer’s instructions. Primer pairs J/L or K/L were used for the construction of ΔN1 or ΔN2 mutants, respectively (**Table 1**). For amplification, an initial denaturation for 2 min at 94°C, followed by 30 cycles consisting of denaturation at 94°C for 30 s, annealing at 63°C for 30 s, and elongation at 68°C for 90 s was performed. Then the PCR products (flanked by *Eco*RI/*Bam*HI restriction sites) were digested by the corresponding enzymes (NEB, Frankfurt (Main)/Germany), ligated into pGEX-6P-1 using T4 DNA Ligase (Promega, Mannheim/Germany) and transformed in One Shot^TM^ TOP-10 *E. coli* strain following manufacturer’s protocol. To prove the correct integration of the amino-terminal deletions, the plasmids were sequenced by GATC Biotech (Konstanz/Germany). The constructs were used for protein overexpression in *E. coli* BL21.

**Table 1:** Primer sequences used for mutagenesis PCR.

Primer	Number	Mutation	Sequence (5′-3′)
A	822F2	H46A/D47A	TAC GCC GCT TCT ATT AAG GAT TCG A
B	822R	H46A/D47A	AGA ATA GAT CGT ATC GTC TTT AG
C	823F2	K50A/D51A	ATT GCG GCT TCG ATT AAA GCG GTG
D	823R	K50A/D51A	GCT AGA TAA GAA TGG TGC TAA GA
E	924F	H46A/D47A/K50A/D51A	ATT GCG GCT TCG ATT AAA GCG GTG GTG
F	924R	H46A/D47A/K50A/D51A	AGA AGC GGC GTA AGA ATA GAT CGT ATC G
G	939F1	ΔN2	GAT TAA AGC GGT GGT GAA TAT CTC TAC TG
H	939R	ΔN2	GAT GCA GCA AAT AAA GCA CTT GCT AAA C
J	935F	pGEX_ΔN1	CCG CTG GGA TCC TCT ATT AAG GAC TC
K	935F1	pGEX_ΔN2	CCG CTG GGA TCC TCT ATT AAG GCG GTG
L	935R	pGEX_ΔN1/2	CGA CCC GGG AAT TCT CAT TTC ACC AAA ATG

The *htrA* complementation construct consisting of the *htrA* gene of *H. pylori* G27 wt, the *htrA* signal peptide from the *C. jejuni* strain 81–176 and *aph3* gene as kanamycin antibiotics resistance marker was cloned as described (Boehm et al., 2017). For the mutagenesis of the amino-terminal cleavage sites, we performed PCRs using Platinum^TM^ *Taq* DNA Polymerase according to manufacturer’s instructions (Thermo Fisher Scientific). The sequences of the used mutagenesis primers are shown in **Table 1**. For the mutagenesis of H46/D47A (primer pair A/B) or K50A/D51A (primer pair C/D), respectively, an initial denaturation step at 94°C for 2 min, followed by 30 cycles each with denaturation at 94°C for 30 s, annealing at 58°C for 30 s, and elongation at 68°C for 7 min was performed. The amplification protocol ends with a final elongation step at 68°C for 10 min. Regarding the mutagenesis of H46A/D47A/K50A/D51A, primer pair E/F was used, the denaturation time was changed to 20 s and the annealing temperature was adjusted to 61°C. For the deletion of amino-terminal sequences (aa G19-D51) primer pair G/H was applied, the denaturation time was reduced to 10 s, the annealing temperature adjusted to 68°C, and the elongation time reduced to 3 min 30 s. Subsequently, the PCR products were subjected to digestion by *Dpn*I (NEB), fill-in-reaction by DNA Polymerase I, Large Fragment (NEB) and re-ligated using T4 DNA Ligase (Promega) following manufacturer’s instructions. To confirm the mutations, the constructs were sequenced. The used sequencing primer is also shown in **Table 1**. Finally, the complementation constructs were transformed into *C. jejuni* 81–176Δ*htrA* deletion mutant as described (Boehm et al., 2015). The correct integration into the chromosome of *C. jejuni* 81–176Δ*htrA* was confirmed by re-amplification using PCR and sequencing. Moreover, the expression of HtrA*^Hp^* in *C. jejuni* 81–176 was confirmed by Western Blotting. Further, the *C. jejuni* Δ*htrA*/*htrA^Hp^* variants were used for analyzing protein secretion and proteolytic activity.

### HtrA Secretion Assay

For secretion of HtrA proteins into the supernatant, *C. jejuni* wt, isogenic Δ*htrA* deletion mutant and Δ*htrA*/*htrA^Hp^* variants were suspended in BHI medium (Oxoid) supplemented with the mutant-specific antibiotics or without, respectively. For monitoring the secretion of HtrA, the bacteria were grown starting at OD_600nm_ = 0.3 and shaking at 160 rpm. When the culture reached an OD_600nm_ between 0.8 and 0.9, the cellular and secreted proteins were separated by centrifugation at 1,500 × *g* for 10 min. The supernatants were again centrifuged for 15 min at 17,000 × *g* to remove cell debris and sterile filtered (Brisslert et al., 2005; Boehm et al., 2012). Bacterial pellets (cellular proteins) and supernatants (secreted proteins) were used for Western Blotting or casein zymography, respectively. All secretion assays were done at least in triplicates.

### Antibodies

Polyclonal antibodies against *C. jejuni* HtrA and CiaB were characterized previously (Boehm et al., 2015). Moreover, we generated a polyclonal antibody against HtrA*^Hp^* using the recombinant protein as antigen. Two rabbits each were immunized using standard protocols by BioGenes GmbH (Berlin/Germany). Immunization was carried out in accordance with German Tierschutzgesetz and Tierschutz-Versuchsverordnung as implementation of the EU directive 2010/63/EU. The protocol was registered and approved by Landesamt für Landwirtschaft, Lebensmittelsicherheit und Fischerei Mecklenburg-Vorpommern (LALLF M-V, Rostock/Germany). All sera were affinity-purified (BioGenes GmbH). The specificity against HtrA*^Hp^* and cross-reactivity with *C. jejuni* HtrA (HtrA*^Cj^*) were verified by Western Blotting (Boehm et al., 2017). For detection of GST, the goat polyclonal antibody against GST was applied (GE Healthcare, Freiburg/Germany).

### Overexpression of HtrA in *E. coli*

For the overexpression of the GST-tagged *H. pylori* 26695 HtrA variants, the constructs (see mutagenesis of *H. pylori htrA*) have been transformed into *E. coli* BL21 (NEB). Briefly, the overexpression was induced by 0.1 mM isopropyl β-D-1-thiogalactopyranoside (IPTG) as described (Löwer et al., 2008). After 4 h induction, the bacteria were harvested at 1,500 × *g* for 15 min. The protein overexpression was analyzed by standard Coomassie staining and Western Blotting.

### SDS–PAGE and Western Blotting

Bacterial cell pellets were adjusted to equal amounts by adding 1× SDS buffer consisting of 62.6 mM Tris–HCl, 2% sodium dodecyl sulfate, 0.01% bromophenol blue, 5% glycerin and reducing agent (Thermo Fisher Scientific), while the supernatants were mixed with 4× SDS buffer. Prior to the separation by SDS–PAGE on 10% polyacrylamide gels, the protein samples were boiled for 10 min at 94°C (Moese et al., 2001). The separated proteins were then analyzed by staining with Coomassie Brilliant Blue R-250 (Bio-Rad, Munich/Germany). For Western Blotting, the proteins were blotted onto PVDF membranes (Immobilon-P, Merck Millipore, Darmstadt/Germany) and blocked in TBS-T buffer (pH 7.4, 0.2 M Tris, 1.4 M sodium chloride and 1% Tween-20) containing 5% milk powder for 1 h at room temperature or at 4°C overnight (Boehm et al., 2011). After addition of the primary antibodies for 2 h at room temperature, horseradish peroxidase-conjugates anti-rabbit polyvalent sheep immunoglobulin was applied as secondary antibody (Zhang et al., 2015). Antibody detection was performed using 1.41 mM luminol in 0.1 Tris–HCl (pH 6.8) supplemented with 0.61 mM p-coumaric acid solved in DMSO and 0.02% hydrogen peroxide. Unless otherwise indicated, all chemicals were obtained from Carl Roth (Karlsruhe/Germany).

### Casein Zymography

Bacterial cell pellets were adjusted to equal amounts by adding phosphate buffered saline and separated in polyacrylamide gels supplemented with 0.1% casein. The gels were renatured in 2.5% Triton-X-100 and equilibrated in developing buffer as described previously (Hoy et al., 2010, 2012). To maintain proteolytic cleavage of casein, the gels were incubated in developing buffer at 37°C overnight. Finally, the caseinolytic activity was visualized using 0.5% Coomassie Brilliant Blue R-250 (Bio-Rad).

### Quantification of Signals in Western Blotting

To investigate the protein expression, band signals on immunoblots were quantified using the Image Lab software (Bio-Rad). The control band was set as 1 and differences are shown as relative amounts of secreted HtrA*^Hp^*. For statistical comparison, Mann–Whitney test using GraphPad Prism 5.01 Software (La Jolla, CA/United States) was performed. Statistical significance was defined as ^∗^*p* < 0.05.

### Immunofluorescence Staining

Polarized Caco-2 cells (ATCC HTB-37) obtained from human colon adenocarcinoma were cultured in RPMI-1640 medium (Invitrogen, Karlsruhe/Germany). The cells were seeded at a concentration of 1.0 × 10^5^ cells in 12-well plates and grown for 72 h. For infection, *C. jejuni* isolates were harvested in BHI medium and the bacterial numbers were determined as optical density (OD) at 600 nm using a spectrophotometer (Eppendorf, Hamburg/Germany). Infections were performed at a multiplicity of infection (MOI) of 50. Twenty four hours post-infection, the cells were fixed and subjected to immunofluorescence staining as described (Harrer et al., 2017). Antibodies against the extracellular domain of E-cadherin (CD324; BD Biosciences, San Jose, CA/United States) and *C. jejuni* (Dako, Glostrup/Denmark) were applied. As secondary antibodies, TRITC (tetramethylrhodamine isothiocyanate)-conjugated goat anti-rabbit and FITC (fluorescein isothiocyanate)-conjugated goat anti-mouse (Thermo Fisher Scientific, Darmstadt/Germany) were used. The specimens were analyzed by the Leica DMI4000B fluorescence microscope with different lasers (Leica Microsystems, Wetzlar/Germany). The images were obtained by LAS AF computer software (Leica Microsystems, Wetzlar/Germany) and optimized in contrast and brightness using Image J-win64 version 2.0. For each condition, representative images are shown.

### Three-Dimensional Modeling of HtrA

The structure of trimeric HtrA*^Hp^* was modeled using the homologous structure DegS from *E. coli* (PDB: 4RQY; de Regt et al., 2015). The homologous region (37% sequence identity; 67% sequence similarity) covers the amino acid residues 36–369 of HtrA*^Hp^*, with the exception of the residues 69–93 that have no equivalent in the template structure. Therefore, modeling was restricted to amino acid residues 36–68 and 94–369. Modeller 9.16 (Webb and Sali, 2014) was used for modeling and RasMol for structure analysis and visualization (Sayle and Milner-White, 1995). The quality of the model was assessed using RAMPAGE (Lovell et al., 2003) and WHATCHECK (Hooft et al., 1996). The resulting model exhibited a good backbone geometry (98% of all residues in the favored regions of the Ramachandran Plot) and no steric clashes larger 0.35 Å were observed.

## Results

### Identifying Amino-Terminal Cleavage Sites in HtrA of *H. pylori*

**FIGURE 1:**
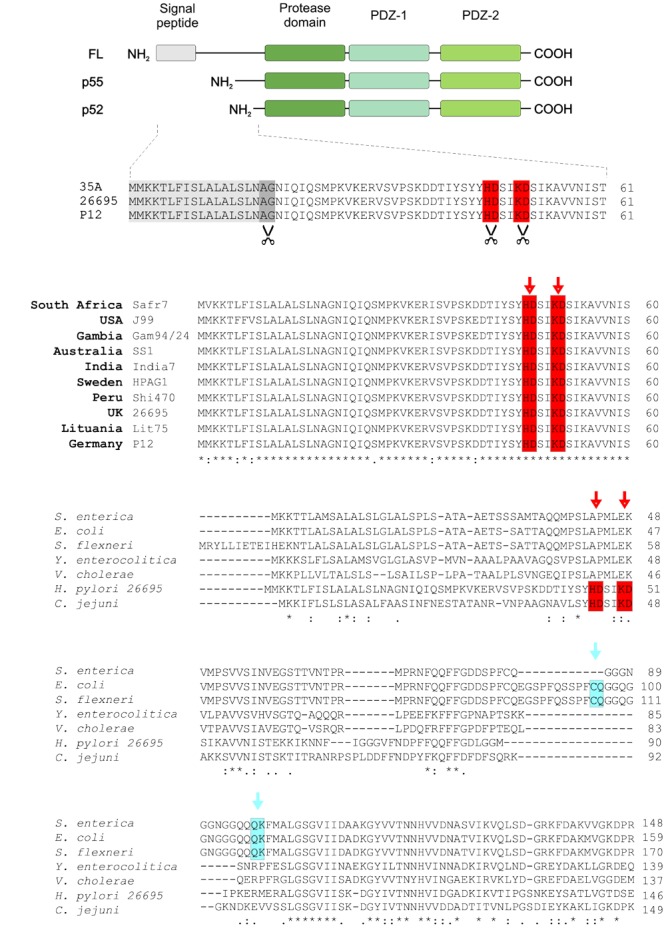
The p55 and p52 forms of HtrA*^Hp^* are generated by amino-terminal cleavage and are conserved in worldwide *H. pylori* strains. **(A)** The domain structures of full-length (FL), p55 and p52 HtrAs are shown. The amino-terminal auto-processing sites, which lead to the generation of p55 and p52 forms, were identified by Edman sequencing in HtrA*^Hp^* of *H. pylori* strains 35A, 26695, and P12. In addition to the cleaved signal peptide (gray box), cleavage sites between H46/D47 and K50/D51 (highlighted with red and scissors), respectively, were determined. **(B)** The amino-terminal HtrA sequences of worldwide *H. pylori* strains were aligned using Clustal Omega, showing that the cleavage sites H46/D47 and K50/D51 are highly conserved (red boxes). The exact cleavage positions are marked by arrows. **(C)** Furthermore, the amino-terminal protein sequence of HtrA*^Hp^* was aligned to that of HtrAs from other Gram-negative gastrointestinal pathogens, revealing that the above cleavage sites (red boxes) are unique for *H. pylori* and *C. jejuni*. However, for the HtrA homologs from *S. enterica, E. coli*, and *S. flexneri* other amino-terminal auto-processing sites at C69/Q70 and Q82/K83 were found (marked with blue).

Analysis of HtrA*^Hp^* protein expression in various *H. pylori* strains revealed that the monomer formed a double-band under reducing conditions, called p55 and p52 (Tegtmeyer et al., 2016). To identify the cleavage sites in HtrA*^Hp^*, protein samples of *H. pylori* strains 35A, 26695, and P12 were subjected to Edman sequencing. Besides the predicted signal peptide, we identified additional amino-terminal cleavage sites between the amino acids at positions H46/D47 or K50/D51, respectively, giving rise to the p55 and p52 HtrA*^Hp^* forms (**Figure 1A**). The characteristic HtrA*^Hp^* double-band was also seen after purification of recombinant HtrA*^Hp^* from *E. coli* (Tegtmeyer et al., 2016), suggesting auto-processing of the protease.

Next, we investigated the conservation of these amino-terminal cleavage sites in HtrA*^Hp^* from *H. pylori* isolates collected in various geographical areas of the world. For this purpose, HtrA*^Hp^* protein sequences of several *H. pylori* strains with African, European, Asian, American, and Australian origin were aligned using Clustal Omega (McWilliam et al., 2013). We found that the processing sites between the amino acid positions H46/D47 and K50/D51 are highly conserved within all investigated *H. pylori* isolates (**Figure 1B**). Moreover, multiple sequence alignment of HtrA*^Hp^* with HtrA protein sequence from several other Gram-negative gastrointestinal pathogens revealed that the identified cleavage sites are present in *H. pylori* and *C. jejuni*, but not in other Gram-negative pathogens including *Escherichia, Salmonella, Shigella, Yersinia*, and *Vibrio* species (**Figure 1C**). These results led us to propose that the highly conserved amino-terminal processing sites of HtrA*^Hp^* might have an important function in *H. pylori.*

### Amino-Terminal Cleavage Affects the Secretion of HtrA^Hp^

To investigate a potential regulatory role of the amino-terminal auto-processing sites on HtrA*^Hp^* secretion, we next studied the involved sequences using the SignalP and SecretomeP databases, predicting a possible secretion based on the signal peptide (Signal P) or a non-classical secretion (SecretomeP), respectively (Bendtsen et al., 2005; Petersen et al., 2011). Using the modified amino acid sequence of HtrA*^Hp^*, we obtained that mutation of the amino-terminal cleavage sites has no effect on the protein secretion (**Table 2**).

**Table 2:** Amino-terminal auto-processing sites of HtrA*^Hp^* appear to have no effect on protein secretion.

	SignalP-value^x^ (Signal peptide based secretion)	SecP-value^∗^ (Non-classical secretion)
HtrA*^Hp^* H46A	0.644	0.555924
HtrA*^Hp^* D47A	0.644	0.561793
HtrA*^Hp^* K50A	0.644	0.547661
HtrA*^Hp^* D51A	0.644	0.567500
HtrA*^Hp^* H46A/D47A	0.645	0.567658
HtrA*^Hp^* K50A/D51A	0.645	0.559475
HtrA*^Hp^* H46A/D47A/K50A/D51A	0.645	0.576967
HtrA*^Hp^*ΔN1	0.633	0.594396
HtrA*^Hp^*ΔN2	0.659	0.547773

*Signal P and SecretomeP databases were used to predict a possible effect of the amino-terminal cleavage sites of HtrA^*Hp*^ on the protein secretion. As template the modified amino acid sequence of HtrA from H. pylori G27 was applied. ^*x*^SignalP-value above 0.57 indicates a possible protein secretion. ^∗^SecP-value above 0.50 indicates a possible protein secretion.*

*HtrA^Hp^* is an essential gene in *H. pylori* and cannot be mutagenized (Salama et al., 2004; Tegtmeyer et al., 2016). We therefore applied the recently established genetic complementation system in *C. jejuni*, where we can express the *htrA* gene of *H. pylori* strain G27 (*C. jejuni*Δ*htrA*/*htrA^Hp^*) to investigate the importance of the above identified amino-terminal cleavage sites on HtrA*^Hp^* secretion experimentally (Boehm et al., 2017). For this purpose, various point and deletion mutations of the amino-terminal cleavage sites were generated (Supplementary Figure 1A). The mutated *htrA^Hp^* gene variants were then transformed into the Δ*htrA* deletion mutant of *C. jejuni* strain 81–176. Correct integration of modified *htrA^Hp^* in the *C. jejuni* 81–176 chromosome was confirmed by PCR and standard sequencing (data not shown). Expression of the modified HtrA*^Hp^* proteins in *C. jejuni* was verified by immunoblotting.

**FIGURE 2:**
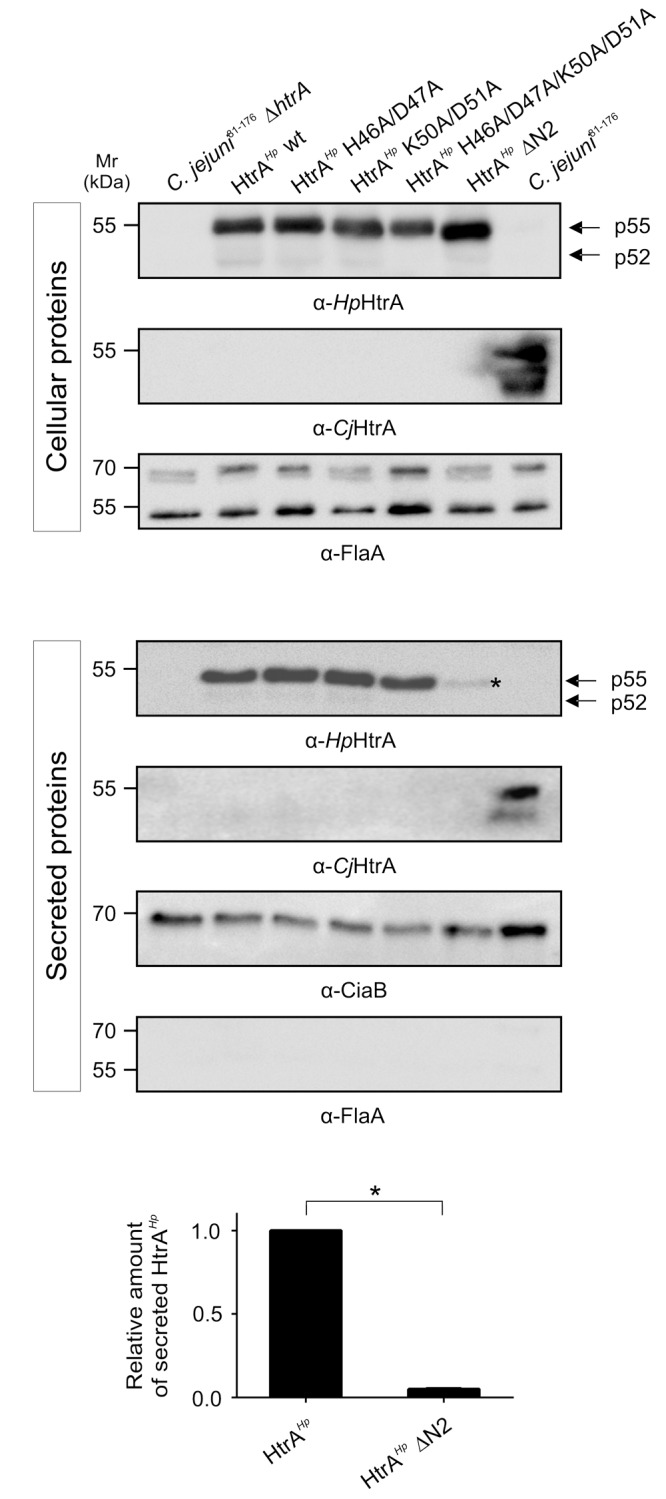
*Helicobacter pylori* HtrA auto-processing sites might affect HtrA*^Hp^* secretion in *C. jejuni* strain 81–176. Point and deletion mutations of the HtrA*^Hp^* amino-terminal cleavage sites (compare Supplementary Figure 1A) were generated and expressed in the Δ*htrA* mutant of *C. jejuni* strain 81–176. In addition to the amino-terminal mutants, Δ*htrA*, Δ*htrA/htrA^Hp^* wt complementant and *C. jejuni* 81–176 wt were grown in BHI liquid broth medium. **(A)** The cellular proteins were subjected to immunoblotting analyzing HtrA*^Hp^*. All samples revealed the expression of the HtrA*^Hp^* monomer as p55 or p52 forms (indicated by arrows). Detection of HtrA*^Cj^* and FlaA expression served as control. **(B)** In addition, secreted proteins were immunoblotted for the presence of HtrA*^Hp^*, and as control for HtrA*^Cj^*, FlaA, and CiaB. The HtrA*^Hp^* p55 or p52 forms (indicated by arrows) were found in the wt complemented and three point mutant strains, while the secretion of HtrA*^Hp^*ΔN2 was strongly decreased (asterisk). **(C)** The band intensities of secreted HtrA*^Hp^* were quantified densitometrically and the relative amount of secreted protein is given. Significant differences were analyzed using Mann–Whitney test (^∗^*p* < 0.05). All secretion assays were performed in triplicates.

To investigate if the amino-terminal cleavage sites have an effect on the protein secretion, the above-described HtrA*^Hp^* variants were grown in BHI liquid broth medium. Secreted and cellular proteins were analyzed by immunoblotting using an antibody against HtrA*^Hp^* (**Figures 2A,B**). All corresponding mutants showed a strong and similar expression of HtrA*^Hp^*, while the Δ*htrA* deletion mutant and the *C. jejuni* 81–176 wild-type (wt) did not (**Figure 2A**). Hence, for the Δ*htrA* mutant no secretion was noted, whereas the Δ*htrA*/*htrA^Hp^* revealed profound HtrA*^Hp^* secretion as expected (**Figure 2B**). However, the wt complementant and variants with only one mutated amino-terminal cleavage site (HtrA*^Hp^* H46A/D47A or HtrA*^Hp^* K50A/D51A) exhibited strong HtrA*^Hp^* secretion levels, while mutation of both cleavage sites resulted in decreased HtrA*^Hp^* secretion (**Figure 2B**). An HtrA*^Hp^* variant missing the entire amino-terminus including both cleavage sites (HtrA*^Hp^*ΔN2) revealed only residual secretion levels, suggesting that the amino-terminus plays a role in the secretion, but not in the general expression of HtrA*^Hp^* (**Figure 2B**, asterisk). As control, probing of the cellular and secreted protein fractions with an antibody against HtrA*^Cj^* revealed strong expression and secretion for the *C. jejuni* 81–176 wt, but not for Δ*htrA* and the Δ*htrA/htrA^Hp^* mutants as presumed (**Figures 2A,B**). Moreover, using an antibody against FlaA, a protein known to be not secreted, we were able to show that the supernatant was free of cell debris and cellular proteins, thus excluding artificial lysis of the bacteria (**Figure 2B**). As FlaA is expressed in the cellular fraction of all samples equally well, we showed that similar amounts of protein were present (**Figure 2A**). As another control, detecting the *Campylobacter* invasion antigen B (CiaB) as a well-known secreted protein (O Cróinín and Backert, 2012) approved that equal amounts of secreted proteins were present, and thus, the differences in HtrA*^Hp^* secretion resulted from mutating the HtrA*^Hp^* amino-terminal cleavage sites (**Figure 2B**).

To quantify HtrA*^Hp^* protein secretion levels upon deleting the HtrA*^Hp^* amino-terminus, the relative amounts of secreted HtrA*^Hp^* were determined. Quantification of secreted HtrA*^Hp^* levels revealed significant lower levels for HtrA*^Hp^*ΔN2 compared to the Δ*htrA/htrA^Hp^* wt complementant (**Figure 2C**), underlying the importance of the amino-terminus for HtrA*^Hp^* delivery into the extracellular environment.

### Amino-Terminal Cleavage Is Involved in Regulating the Proteolytic Activity of HtrA^Hp^

Next, we aimed to investigate if amino-terminal cleavage affects the proteolytic activity of HtrA*^Hp^*. To test this idea, cellular proteins of the above-described HtrA*^Hp^* variants expressed in the *C. jejuni* 81–176 Δ*htrA*/*htrA^Hp^* complementation system were subjected to casein zymography. First of all, in *C. jejuni* only the p55 HtrA*^Hp^* form showed proteolytic in-gel activity, while the p52 form did not (Supplementary Figure 2). However, *C. jejuni* 81–176 wt showed strong caseinolytic active HtrA trimers (arrow). Moreover, for the cellular proteins of HtrA*^Hp^*ΔN2 no proteolytic activity was found and the H46A/D47A/K50A/D51A mutant was strongly reduced (yellow asterisk), while the other HtrA*^Hp^* variants carrying single point mutations in the amino-terminal cleavage sites, the wt complementant and *C. jejuni* wt showed strong caseinolytic active HtrA monomers. As control, no activity was detected for the Δ*htrA* deletion mutant as expected (Supplementary Figure 2). Together, these data suggest a role of the HtrA*^Hp^* amino-terminus in regulating/maintaining the proteolytic activity of this enzyme.

**FIGURE 3:**
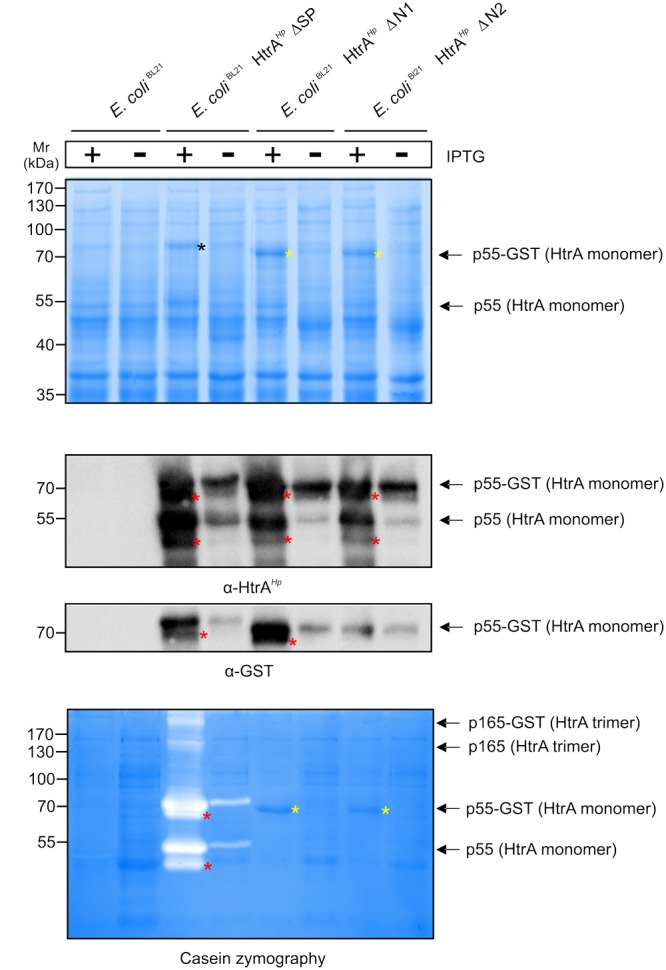
Importance of auto-processing for oligomerization and caseinolytic activity of HtrA*^Hp^* expressed in *E. coli*. The amino-terminus of HtrA*^Hp^* was mutagenized by generating deletion variants and expressed as GST-tagged variants in *E. coli* BL21 (compare Supplementary Figure 1B). *E. coli* BL21 expressing GST-tagged HtrA*^Hp^* without the signal peptide (ΔSP) and empty vector *E. coli* BL21 were used as controls. **(A)** After induction of protein expression by IPTG, the protein lysates were subjected to Coomassie staining. Overexpression of the HtrA*^Hp^* p55 monomers and the GST-tagged variants was observed (arrows). The amino-terminal deletion variants (ΔN1 and ΔN2, yellow asterisks) exhibit a lower molecular weight compared to HtrA*^Hp^*ΔSP (black asterisk). **(B)** The bacterial lysates were subjected to immunoblotting against HtrA*^Hp^* and GST. Overexpression of the GST-tagged HtrA*^Hp^* p55 variants was confirmed by detecting the HtrA*^Hp^* or GST protein, respectively (arrows). In addition, immunoblotting against HtrA*^Hp^* showed the presence of HtrA*^Hp^* monomers without GST-tag (arrow). The p52 form is migrating slightly below p55 (red asterisks). **(C)** Finally, the bacterial lysates were analyzed by casein zymography. For wt HtrA*^Hp^*ΔSP, a strong caseinolytic activity of the HtrA*^Hp^* monomers and its GST-tagged variants was observed (arrows). The p52 form is migrating slightly below p55 (red asterisks). Moreover, caseinolytic activity for the HtrA*^Hp^* trimer and its GST-tagged variant was shown (arrows). The ΔN1 and ΔN2 variants revealed no proteolytic activity (yellow asterisks). All assays were done in triplicates.

To investigate the importance of the amino-terminus on the proteolytic activity of HtrA*^Hp^* in more detail, wt HtrA*^Hp^*, ΔN1 or ΔN2 were expressed as GST-tagged variants in *E. coli* strain BL21 (Supplementary Figure 1B). *E. coli* BL21 were induced for 4 h with IPTG and the resulting bacterial lysates were subjected to Coomassie staining, confirming that equal amounts of protein were present (**Figure 3A**). HtrA*^Hp^* wt missing only the signal peptide (ΔSP) revealed a strong overexpression of the fusion protein (p55 HtrA*^Hp^* with GST-tag of ∼70 kDa) as detected by Coomassie-staining (**Figure 3A**, black asterisk). Moreover, a slight expression of the HtrA*^Hp^* monomer (55 kDa) without GST-tag was found (**Figure 3A**, arrow). Similar expression patterns were observed for the amino-terminal deletion variants of HtrA*^Hp^*, ΔN1, and ΔN2. These modified variants have only a slightly lower molecular weight compared to the wt HtrA*^Hp^* (ΔSP) resulting from the loss of the amino-terminus (**Figure 3A**, yellow asterisks). In addition, bacterial lysates were subjected to immunoblotting using antibodies against the HtrA*^Hp^* protein or GST, respectively (**Figure 3B**). Detection of HtrA*^Hp^* confirmed the strong overexpression of the p55 HtrA*^Hp^* monomer, with or without GST-tag (**Figure 3B**, arrows). Additionally, similar expression patterns were obtained for the p52 forms (**Figure 3B**, red asterisks). As control, *E. coli* BL21 without a plasmid exhibited no expression of HtrA*^Hp^* or GST-tagged protein, respectively (**Figures 3A,B**). Together, these findings confirmed the successful overexpression of HtrA*^Hp^* variants in *E. coli* BL21, useful for further analysis of HtrA activity.

As next, the above generated *E. coli* BL21 lysates were subjected to casein zymography to investigate an effect of the shortened amino-terminus of HtrA*^Hp^* on the proteolytic activity. Wt HtrA*^Hp^* (ΔSP) showed strong caseinolytic bands at about 70 and 55 kDa resulting from the fusion protein of HtrA*^Hp^* with GST-tag or the HtrA*^Hp^* protein without tag, respectively (**Figure 3C**, arrows). Reduced proteolytic activity was also detected for the corresponding wt p52 HtrA*^Hp^* monomers, with and without GST-tag (**Figure 3C**, red asterisks). In addition, caseinolytic active bands at about 210 and 165 kDa were found, corresponding to the HtrA*^Hp^*ΔSP trimer with or without GST tag, respectively (**Figure 3C**, arrows). Importantly, both HtrA*^Hp^* variants missing the amino-terminus (ΔN1 and ΔN2) revealed no caseinolytic activity, albeit the proteins are strongly expressed (**Figure 3C**, yellow asterisks). These experiments demonstrate that deletion of the amino-terminus resulted in abrogation of HtrA*^Hp^* proteolytic activity.

### Modifications Within the Amino-Terminus of HtrA^Hp^ Affect Disruption of Cell-to-Cell Junctions

**FIGURE 4:**
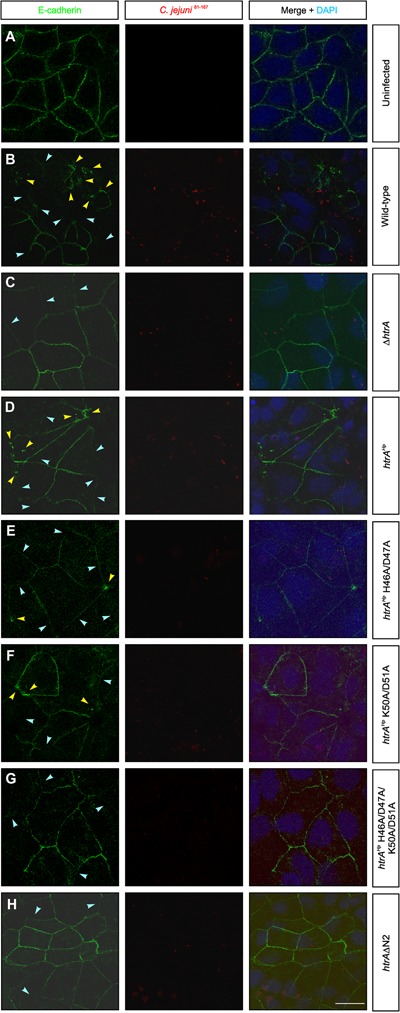
Amino-terminal auto-processing of HtrA*^Hp^* influences the disruption of cell-to-cell junctions during infection with *C. jejuni*. Polarized Caco-2 cells were **(A)** left uninfected (mock) or infected for 24 h with **(B)** *C. jejuni* 81–176 wt, **(C)** Δ*htrA* or **(D)** Δ*htrA/htrA^Hp^* wt. **(E–H)** Infection with *C. jejuni* 81–176Δ*htrA* expressing point and deletion mutants of HtrA*^Hp^* amino-terminal auto-processing sites (compare Supplementary Figure 1A). The cells were subjected to immunofluorescence staining detecting E-cadherin (green) and *C. jejuni* (red). Arrowheads mark cells showing significantly downregulated (blue) or locally disrupted (yellow) E-cadherin signals. The nuclei were stained with DAPI (blue). The scale bar corresponds to 10 μm.

As changes within the amino-terminus interfere with the proteotytic activity of HtrA*^Hp^*, we aimed to study the outcome during infection. For this purpose, confluent polarized Caco-2 cells were infected with the above-characterized *C. jejuni* 81–176Δ*htrA/htrA^Hp^* variants and corresponding control strains for 24 h. Subsequently, the cells were fixed and subjected to immunofluorescence staining using antibodies against the adherens junction protein E-cadherin and *C. jejuni*. We could confirm that the signals of the *C. jejuni* bacteria being attached to the host cells were equally high between the infected samples, while the mock control cells showed no bacteria as expected (red, **Figure 4**). Moreover, while the uninfected mock control cells exhibited the typical E-cadherin signals between the neighboring cells in the monolayer, infection with *C. jejuni* wt and the Δ*htrA/htrA^Hp^* complementant led to a significant disruption of the E-cadherin staining in a HtrA-dependent fashion (green, **Figures 4A–D**). Individual cells showing downregulated or mislocated E-cadherin signals are marked with blue and yellow arrowheads, respectively. Infection with strains expressing HtrA*^Hp^* carrying mutations in the single cleavage sites H46A/D47A or K50A/D51A, respectively, also resulted in the downregulation and mislocalization of E-cadherin signals, but less pronounced (**Figures 4E,F**). In contrast, the E-cadherin patterns were still widely intact during infection with the HtrA*^Hp^* double mutant H46A/D47A/K50A/D51A or HtrA/HtrA*^Hp^*ΔN2 deletion variant, respectively, suggesting an important role of amino-terminal HtrA*^Hp^* cleavage at both sites on protease activity, and thus, on damaging cell-to-cell junctions (**Figures 4G,H**).

### Modeling the Structure of the Trimeric HtrA

**FIGURE 5:**
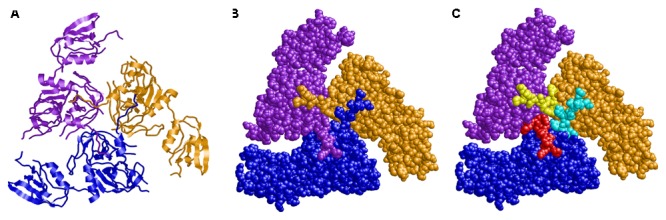
Three-dimensional modeling of trimeric HtrA of *H. pylori*. The subunits are shown in **(A)** ribbon presentation and **(B,C)** space–filled presentation and colored in blue, purple, and orange, respectively. **(C)** The amino–terminal residues 36–46, which are removed by proteolytic cleavage, are highlighted in different colors (cyan, red, and yellow) for the three individual subunits. The amino–termini form tight interactions with the adjacent subunits thereby interlocking the trimeric conformation.

Finally, we aimed to investigate the potential importance of HtrA auto-processing by structural modeling. The model of HtrA*^Hp^* revealed a trimer as known from other HtrA proteins in Gram-negative bacteria. Interestingly, this trimer is stabilized via interactions of the amino-terminal arm (residues 36–45), which protrudes from the protease domain and interacts with the adjacent subunits, thereby interlocking the conformation (**Figures 5A,B**). The intermolecular interactions of this arm are conserved among different members of the HtrA family of proteases including DegP, DegQ, and DegS. We selected DegS as a modeling template because it exhibits the highest local sequence similarity to HtrA*^Hp^* in the amino-terminal region. The model also revealed that the H46/D47 cleavage site is located almost exactly between the N-terminal arm and the globular part of the enzyme. Thus, a cleavage at this position will remove the N-terminal arm, which is responsible for the majority of the inter-subunit interactions (**Figure 5C**). The lack of these interactions will drastically reduce trimer stability and is expected to result in the dissociation of the subunits.

## Discussion

The serine protease HtrA is a conserved periplasmic protein and has an important impact on the virulence and survival of various Gram-negative pathogens (Ingmer and Brøndsted, 2009; Frees et al., 2013; Skorko-Glonek et al., 2013). Inactivation of the *htrA* gene and generation of Δ*htrA* knockout mutants have been already described in various bacteria, suggesting that this gene is not essential in the majority of pathogens (Humphreys et al., 1999; Pedersen et al., 2001; Cortés et al., 2002; Purdy et al., 2002; Wilson et al., 2006; Hoy et al., 2010; Gloeckl et al., 2013; Heimesaat et al., 2014a,b), while the inactivation of *htrA^Hp^* in a large number of worldwide *H. pylori* isolates failed (Salama et al., 2004; Tegtmeyer et al., 2016). In addition, recent studies have shown that pharmacological inhibition of the HtrA*^Hp^* activity leads to killing of *H. pylori*, but did not affect the growth of *Salmonella* or *Shigella* species, underlying the essentiality of the gene in *H. pylori* (Tegtmeyer et al., 2016). Here, we discovered and characterized amino-terminal auto-processing events of HtrA*^Hp^*. Our data demonstrate that cleavage of HtrA*^Hp^* appears at specific and conserved sites in worldwide strains, and significantly reduced the stability of trimer formation, and thus affect oligomerization, secretion and regulatory activities of the protease with an important role in the pathogenesis of *H. pylori*.

### Amino-Terminal Cleavage of HtrA^Hp^ and Effects on Trimerization

Analyses of more than one thousand worldwide *H. pylori* strains demonstrated that HtrA*^Hp^* is conserved among these isolates and that the mature full-length HtrA*^Hp^* proteins are expressed as a p55 and p52 double-band (Tegtmeyer et al., 2016). We proposed a role of HtrA*^Hp^* auto-processing for its proteolytic activity and extracellular transport. Using Edman sequencing we identified two amino-terminal cleavage sites at positions H46/D47 or K50/D51 and their cleavage gives rise to the p55 and p52 HtrA*^Hp^* protein forms. Moreover, we could demonstrate that these cleavage sites are highly conserved among *H. pylori* strains originating from different countries worldwide. Interestingly, these sites have been also found in HtrA of the close relative *C. jejuni*, but not in other Gram-negative bacteria. By comparison, for the HtrA homolog DegP from *E. coli* auto-cleavage was also identified, after amino acid position C69 or Q82, respectively (Skórko-Glonek et al., 1995), which is different from the cleavage we observed in HtrA*^Hp^*. Interestingly, HtrA proteins from bacteria and higher organisms can form proteolytically active trimers, and trimerization sequences were reported. For example, it was proposed that the multimerization of *E. coli* DegP occurs via the interactions between Q-linker sequences, a less conserved region in the protease domain (Kim and Kim, 2005). In addition, several mammalian HtrA proteins exhibit the trimerization motif QYNFIA, which is conserved and located at the amino-terminus (Nam et al., 2006). Using deletion variants of this motif in the mitochondrial serine protease HtrA2, trimerization was abrogated (Li et al., 2002). Especially, a phenylalanine residue (F149) in this motif is crucial for HtrA homotrimer formation in mammals (Nam et al., 2006). Regarding the trimerization of HtrA*^Hp^*, our structural model suggested that an amino-terminal arm, comprising the amino acid residues 36–45, plays an important role in mediating the interaction between individual HtrA*^Hp^* monomers, and thus, is able to influence the stability of the trimer. These findings lead us to suggest that the auto-processing sites identified in *H. pylori* have an essential regulatory role and might be crucial for the pathogenesis of *H. pylori* and eventually for *C. jejuni*, which deserves further investigation in prospective studies.

### Mutational Effects on HtrA Secretion and Proteolytic Activity

To reconsider the impact of the amino-terminal cleavage sites, we aimed to investigate the effect on secretion and proteolytic activity in bacteria. Research on the functions of HtrA*^Hp^* is complicated because the gene is essential in *H. pylori* and Δ*htrA* knockout mutants are not available (Salama et al., 2004; Tegtmeyer et al., 2016, 2017). Thus, we decided to apply the recently established genetic complementation system of *htrA* from *H. pylori* strain G27 in *C. jejuni* 81–176 (Boehm et al., 2017). It is well known that HtrA proteins in Gram-negative bacteria including *H. pylori* contain a signal peptide being important for the Sec-dependent transport of the protein across the inner membrane into the periplasm (Clausen et al., 2002; Ingmer and Brøndsted, 2009; Frees et al., 2013; Skorko-Glonek et al., 2013), while its transport across the outer membrane remains unclear. To investigate the proposed roles of the H46/D47 or K50/D51 cleavage sites, we constructed point mutations in both cleavage sites, either separately (H46A/D47A and K50A/D51A) or together (H46A/D47A/K50A/D51A). In addition, we deleted the amino-terminus of HtrA including both cleavage sites (ΔN2) and expressed the HtrA*^Hp^* mutants in *C. jejuni*Δ*htrA* deletion variant. We confirmed equal expression of HtrA*^Hp^* variants, while the secretion was significantly downregulated by the H46A/D47A/K50A/D51A or ΔN2 mutants. However, it is not clear if the amino-terminal cleavage affects the secretion or if the alanine exchange or deletion of the entire amino-terminus, respectively, led to structural changes within the trimer, resulting in a disturbed HtrA*^Hp^* secretion. Moreover, we investigated the role of amino-terminal auto-cleavage on the caseinolytic activity of HtrA*^Hp^*, which is also conserved among hundred worldwide *H. pylori* isolates (Tegtmeyer et al., 2016). Our studies showed that mutation of the amino-terminus including both cleavage sites (ΔN2) resulted in the loss of HtrA activity in the *C. jejuni* complementation system. To further characterize if the reduction of activity resulted from loss of the entire amino-terminus, we created HtrA*^Hp^* variants without the amino-terminus including cleavage site H46/D47 (ΔN1) or H46/D47 and K50/D51 (ΔN2), respectively, and expressed the HtrA*^Hp^* variants heterologously in *E. coli* BL21. As observed in *C. jejuni*, HtrA*^Hp^*ΔN2 showed no proteolytic activity when expressed in *E. coli* BL21. In addition, no caseinolytic activity was detected for the ΔN1 mutant. This might be an effect of a disturbed interaction between the subunits within the HtrA trimer. HtrA*^Hp^* shows no sequence homology to typical auto-transporters, which process themselves by auto-proteolysis (Boehm et al., 2013), indicating that the auto-cleavage sites might not affect the secretion, but can modulate the inter-trimer interactions. Caseinolytic active oligomers with sizes of a trimer and higher were found in multiple tested *H. pylori* isolates from different origins, both in total cell lysates and culture supernatant (Tegtmeyer et al., 2016). In addition, studies on HtrA in *E. coli* have shown that the oligomers are highly proteolytic active compared to the monomer (Krojer et al., 2008). In line with these observations, deletion of the trimerization motif in human HtrA2 showed that the formation of the homotrimer is essential for precise function of the enzyme including its proteolytic activity (Li et al., 2002; Nam et al., 2006). Recently, it was experimentally also shown that trimerization plays a fundamental role for the activation of human HtrA1 by an allosteric mechanism (Cabrera et al., 2017). Further, computational studies of DegS suggest that disassembly of a DegS trimer inhibits proteolytic activity (Lu et al., 2016). Therefore, an altered interaction within the trimers resulting from the loss of the amino-terminus could led to destabilization of the trimer and thus, to the lack of the proteolytic activity of HtrA*^Hp^*.

### Importance of Amino-Terminal HtrA Sequences to Disrupt E-cadherin in Caco-2 Cells

Previous studies revealed that binding of the bacteria to the epithelial host cells depends on the HtrA expression (Brøndsted et al., 2005). Furthermore, secreted HtrA*^Hp^* can cleave the ectodomain of the adherens junction protein E-cadherin in polarized gastric epithelial cell models *in vitro* and *in vivo* (Weydig et al., 2007; Schirrmeister et al., 2009; Hoy et al., 2010, 2012; Boehm et al., 2012; Tegtmeyer et al., 2016). Infecting polarized epithelial cells with our generated HtrA*^Hp^* variants carrying mutations in the amino-terminal cleavage sites showed a lower disruption of the E-cadherin pattern compared to the wt HtrA complementant. This underlines the proposal, that disturbed trimer interaction caused by the missing or modified amino-terminus, respectively, led to an altered proteolytic activity of HtrA*^Hp^*. Thus, the HtrA-dependent disruption of E-cadherin based cell-to-cell junctions is affected. Besides E-cadherin, HtrA*^Hp^* can act directly on the tight junction proteins occludin and claudin-8 (Tegtmeyer et al., 2017). Consequently, an effect of the HtrA*^Hp^* amino-terminal processing on the cleavage of occludin and claudin-8 should be further investigated in prospective studies. Our data suggest that the conserved amino-terminal cleavage sites H46/D47 and K50/D51 are important for the auto-processing of HtrA*^Hp^* and seem to be involved in oligomerization and hence, in regulating activity and secretion of the novel virulence factor HtrA. In line, amino-terminal cleavage being important for trimer stabilization seems to influence the disruption of the epithelial cell barrier by affecting HtrA-dependent E-cadherin cleavage.

## Conclusion

Here, we presented for the first time that the p55 and p52 forms of HtrA*^Hp^* result from amino-terminal cleavage of the protein between amino acid positions H46/D47 or K50/D51, respectively. Moreover, these amino-terminal cleavage sites are conserved within *H. pylori* and *C. jejuni*. Three-dimensional modeling of HtrA showed that the amino-terminus of HtrA*^Hp^* might be essential for oligomerization of HtrA monomers and stabilization of the trimer. Point and deletion mutants of these identified amino-terminal processing sites were constructed to investigate effects on protein secretion or activity using Western Blotting or Casein zymography, respectively. Interestingly, loss of the entire amino-terminus including these cleavage sites could lead to strong structural changes within the trimer being deleterious for the activity. Lack of certain interactions within the trimer caused by mutagenesis of the amino-terminal cleavage sites might also led to destabilization of the trimer and a lower enzymatic efficiency and protein transport. In addition, infection of Caco-2 cells confirmed that the loss of entire amino-terminus led to a disturbed E-cadherin shedding, perhaps caused by a changed proteolytic activity as result of an altered oligomerization. Thus, this study provides a basis for further analysis to investigate the role of amino-terminal cleavage of HtrA for pathogenesis of *H. pylori*.

## Author Contributions

NT and SB conceptualized the study. NA, NT, and HS performed the experiments and generated the data. NT, SB, NA, HS, and JS-G analyzed and interpreted the data. SB and NA wrote the paper. All authors revised and agreed on the manuscript.

## Conflict of Interest Statement

The authors declare that the research was conducted in the absence of any commercial or financial relationships that could be construed as a potential conflict of interest.
