# Supplementary material for: Amino-Terminal Processing of Helicobacter pylori Serine Protease HtrA: Role in Oligomerization and Activity Regulation
Source: Front Microbiol. 2018 Apr 16;9:642. doi: 10.3389/fmicb.2018.00642 (PMC5911493; doi:10.3389/fmicb.2018.00642)
Supplement: Supplementary file 1 [file Data_Sheet_1.docx]

Supplementary Material

**Amino-terminal processing of *Helicobacter pylori* serine protease HtrA: role in oligomerization and activity regulation**

Nicole Albrecht, Nicole Tegtmeyer^*^, Heinrich Sticht, Joanna Skórko-Glonek, Steffen Backert

*** Correspondence:** Nicole Tegtmeyer: [nicole.tegtmeyer@fau.de](mailto:nicole.tegtmeyer@fau.de)

# Supplementary Figures

## SUPPLEMENTARY FIGURE 1 │ Point and deletion mutants of HtrA*^Hp^* amino-terminal cleavage sites produced in *C. jejuni* (A) or *E. coli* (B). (A) The *htrA* complementation construct (p*Hp*^G27^HtrA) encoding the wt *htrA* gene of *H. pylori* strain G27, the *htrA* signal peptide from *C. jejuni* strain 81-176 and the kanamycin resistance marker *aph3* was used as template for the generation of HtrA*^Hp^* amino-terminal mutations. Mutagenesis in the cleavage sites H46/D47 and/or K50/D51 was performed by alanine exchange. The amino-terminus including both cleavage sites was also deleted (ΔN2). (B) In addition, using vector pGEX‑6P‑1 encoding GST fusions of the *htrA* gene from *H. pylori* strain 26695 (lacking the signal peptide, ΔSP), the indicated amino-terminal deletion variants of HtrA*^Hp^* were constructed, including the cleavage site H46/D47 (ΔN1) and both cleavage sites, H46/D47 and K50/D51 (ΔN2).

**SUPPLEMENTARY FIGURE 2 │ Amino-terminal cleavage of *H. pylori* HtrA is involved in regulating activity in *C. jejuni* 81-176.** Point and deletion mutations of the HtrA*^Hp^* amino-terminal cleavage sites (compare supplementary figure 1A) were expressed in the *C. jejuni* 81-176 Δ*htrA*/*htrA^Hp^* complementation system. Besides the amino-terminal deletion variants, Δ*htrA*, Δ*htrA/htrA^Hp^* wt complementant and *C. jejuni* 81‑176 wt were grown as control in BHI broth medium. The caseinolytic activity of cellular proteins was investigated by casein zymograpy. In addition to the proteolytic active HtrA*^Hp^* p55 monomer, *C. jejuni*^81-176^ wt revealed the presence of proteolytic active HtrA*^Hp^* trimers (p165) as marked by arrows. Proteolytic active p52 monomers were not seen. An unknown 65 kDa HtrA-independent protease, present in all *C. jejuni* strains, is marked by an asterisk.
