# Supplementary figures and images for: Amino-Terminal Processing of Helicobacter pylori Serine Protease HtrA: Role in Oligomerization and Activity Regulation
Source: Front Microbiol. 2018 Apr 16;9:642. doi: 10.3389/fmicb.2018.00642 (PMC5911493; doi:10.3389/fmicb.2018.00642)

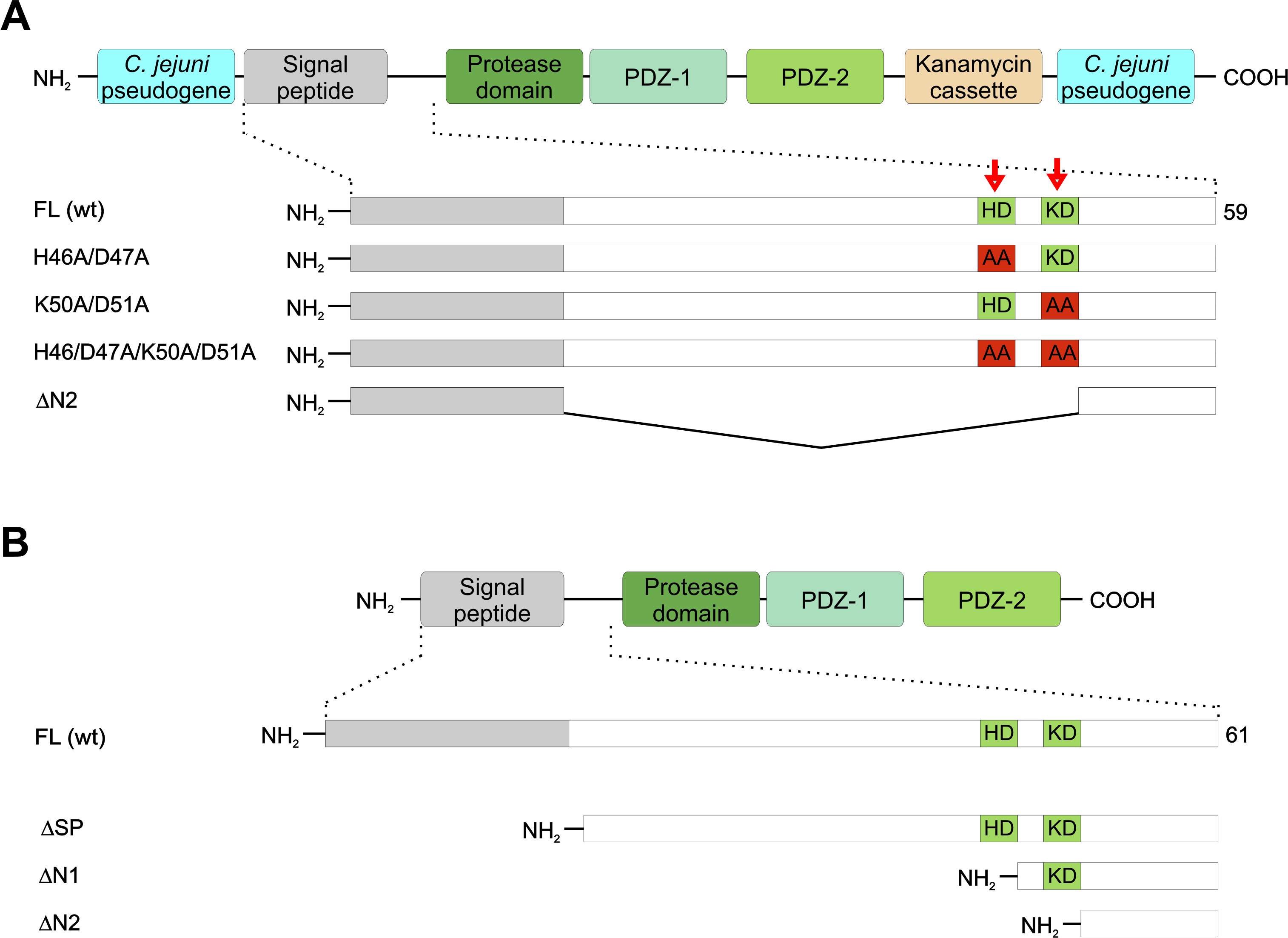

Supplement: Supplementary file 2 [file Image_1.TIF]

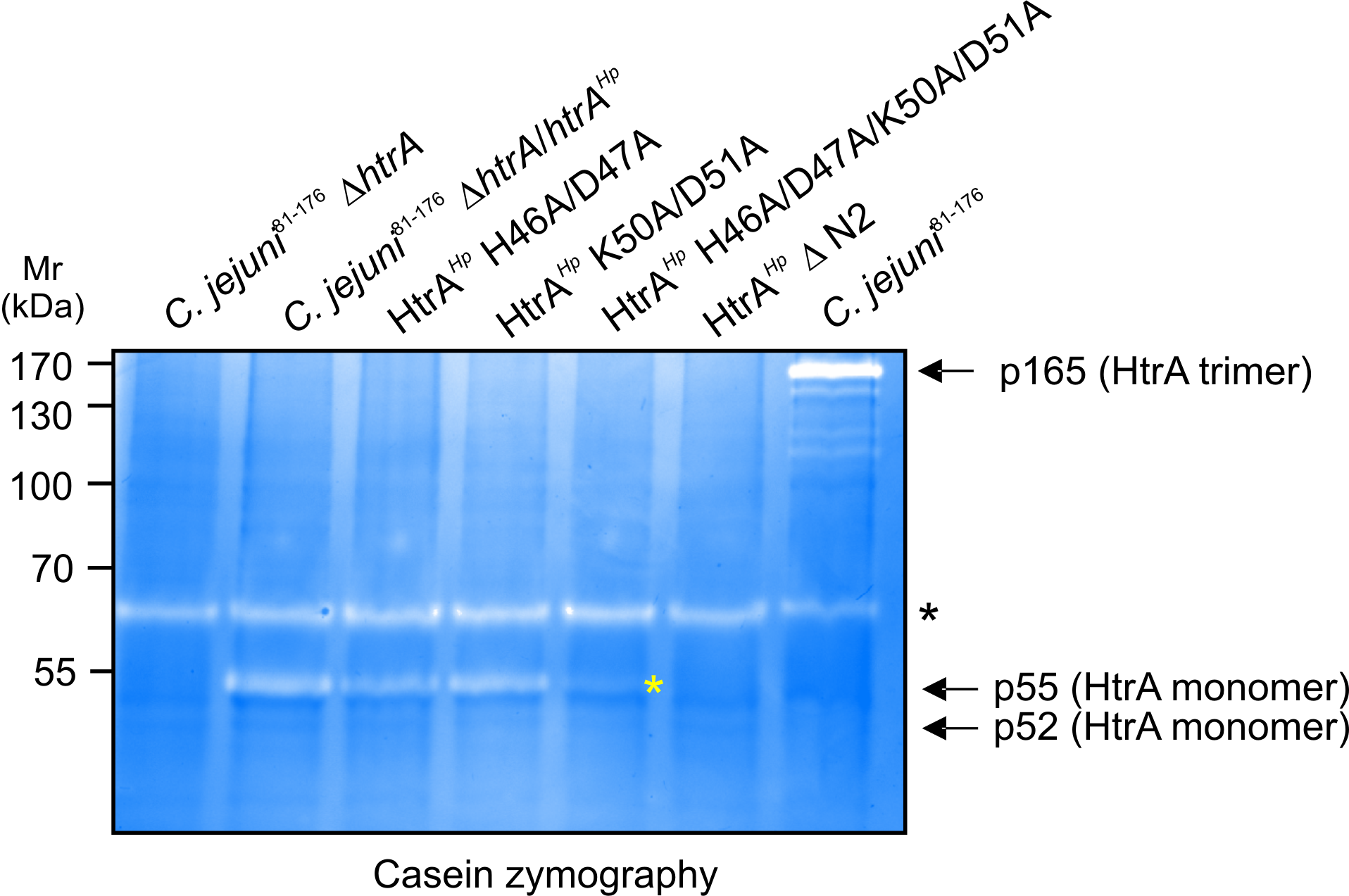

Supplement: Supplementary file 3 [file Image_2.TIF]
